# Introducing a Sustainable Framework for Preschool Visual Acuity Screening: The Alexandroupolis Case

**DOI:** 10.3390/jcm15051907

**Published:** 2026-03-03

**Authors:** Georgios Labiris, Christos Giazitzis, Christina Mitsi, Minas Bakirtzis, Eirini-Kanella Panagiotopoulou, Eirini Vavanou, Aristeidis Konstantinidis, Panagiota Ntonti, Nikolaos Polyzos

**Affiliations:** 1 Department of Ophthalmology, University Hospital of Alexandroupolis, Dragana, 68100 Alexandroupolis, Greece; christos_giazitzis@hotmail.com (C.G.); minas961@hotmail.com (M.B.); giotado@hotmail.com (P.N.); 2 Department of Medicine, Democritus University of Thrace, Dragana, 68100 Alexandroupolis, Greece; npolyzos@med.duth.gr

**Keywords:** amblyopia, visual screening, visual acuity, DDiVAT

## Abstract

**Background/Objectives:** Western societies introduce school-based or school-linked programs in order to improve the physical health status of students and prevent the negative impact of the late diagnosis of a series of diseases and conditions. Preschool visual acuity (VA) screening represents an established school-based approach aimed at the early detection of amblyopia risk factors and vision-related learning difficulties. In this study, we report the methods and outcomes of the first officially organized kindergarten-based VA screening program in Greece, implemented using the Democritus Digital Visual Acuity Test (DDiVAT) screening suite and involving trained educators as part of the screening workflow. The present analysis focuses on the operational performance and screening outcomes within this defined setting. **Methods:** This study was a kindergarten-based screening. Each kindergarten was equipped with the DDiVAT screening framework, which consisted of a 32-inch, 4K, Android Smart TV with the DDiVAT application preinstalled, a site-license granting access to the secure DDiVAT database, and two vouchers for teachers to participate in the official lifelong DDiVAT training program. **Results:** From 2476 enrolled students, 207 (8.36%) were referred due to suboptimal presenting VA in one or both eyes. Average VA ranged from logMAR 0.11 to 0.07, which is consistent with former reports. **Conclusions:** No major technical difficulties were encountered, suggesting that DDiVAT may represent a feasible digital approach for preschool VA screening in real-world educational settings.

## 1. Introduction

It is a truism that published reports have indicated the importance of kindergartens and schools in delivering services beyond the academic realm [1]. Especially during and after the COVID-19 epidemic, Western societies depended heavily on schools in order to improve the physical health status of students and prevent the negative impact of the late diagnosis of numerous diseases and conditions [2]. These school-based or school-linked health interventions include vaccination, oral monitoring, special diets, myopia screening, and others. In fact, screening programs in schools are so popular that they can be found in almost every Western country. The role of teachers in delivering healthcare services has been controversial. Among the advantages of enrolling school staff in screening programs is high acceptance by students and the parents [3], their availability for regular follow-up, and their high specificity rates [4]. On the other hand, poorly trained teachers fail to adhere to the screening methods, resulting in high false-positive numbers, and might neglect their academic duties. Prompt detection of suboptimal visual acuity (VA) in preschool children is essential for their proper development, their academic readiness, and their overall quality of life [5,6]. Uncorrected refractive errors, strabismus, and other ocular conditions often go unnoticed in early childhood, contributing to new cases of amblyopia, reduced school performance, and a reduced number of achievements, particularly in the absence of systematic screening. Despite the importance of preschool vision screening, access remains uneven, particularly in rural or geographically isolated areas [7].

In Greece, no official kindergarten-based program for VA screening in the preschool population is implemented. Instead, preschool ophthalmological screening is performed either by pediatricians or by ophthalmologists, following referral by pediatricians. However, the methods of preschool VA screening in Greece are inconsistent and mostly rely on the parents’ initiative. It is no surprise that Greece publishes no official data regarding the prevalence of amblyopia and refractive errors in the preschool and school populations. The Democritus Digital Visual Acuity Test (DDiVAT) is a validated, certified Class-1 screening framework for the reliable measurement of VA in any remote setting [8]. It comprises: (a) a Smart-TV application that can be installed through the corresponding application store, (b) a secure cloud database, (c) a smart-trapping subsystem for improving the reliability of VA screening. DDiVAT has been used in several official screening programs, among them, for refugees and immigrants [9].

Within this context, the primary objective of the present study is to describe and analytically evaluate the methodology and screening outcomes of the first kindergarten-based VA screening program conducted in Greece, with emphasis on its practical implementation within a real-world educational environment.

## 2. Materials and Methods

### 2.1. Setting

This was a kindergarten-based screening study. The protocol adhered to the tenets of the Helsinki Declaration, and written informed consent was obtained from the legal guardians of all participants. The Research Ethics Committee of the Democritus University of Thrace approved the protocol (DUTH/EHDE2/20-10-2022). The study was conducted in the metropolitan municipality of Alexandroupolis, in Greece, following permission by the Regional Directorate of Primary Education and by the 4th Regional Health Authority, for three consecutive academic years (2022–2025). The official registration number of the study is NCT05835193.

### 2.2. Participants

From a total of 3092 registered preschool students, 2476 (80.1%) participated in the study (1256 boys and 1220 girls) and were divided into three study groups according to their age: study group 1 (SG1) included 962 children aged 36–60 months old, study group 2 (SG2) included 1194 children aged 61–72 months old, and study group 3 (SG3) included 269 children aged 73–84 months. Despite obtaining guardians’ consent, 51 children (2.1%) failed to cooperate, and the examination could not be completed (Table 1).

Regarding ophthalmological history, 42 (1.70%) students used spectacles, 16 (0.65%) had been diagnosed with amblyopia and/or strabismus, and 3 (0.12%) had a history of ocular surgery.

### 2.3. DDiVAT VA Screening Framework

All participating kindergartens were selected from the public kindergarten network of the Metropolitan Municipality of Alexandroupolis following approval from the competent educational and health authorities and written informed consent from the children’s legal guardians. To ensure methodological consistency across screening sites, identical hardware was used in all kindergartens. A DDiVAT screening framework was provided in all kindergartens, which included: (a) a 32-inch, 4K, Android Smart-TV, preinstalled with the DDiVAT application, (b) a site-license to the secure DDiVAT database, and (c) two vouchers for the official DDiVAT lifelong training program for the teachers. VA testing was performed using the DDiVAT application, which incorporates an automatic calibration procedure executed once upon first installation at each site. This calibration ensures standardized testing distance, symbol size scaling, and luminance settings according to manufacturer specifications, after which no manual adjustments by the examiner are required. The lifelong training program for teachers is a 90 h blended course, with distance-learning theoretical lectures on the fundamentals of VA screening in preschool children, and on-site, hands-on training on the optimal DDiVAT operation. Following the training of the teachers and the proper installation of DDiVAT, each kindergarten was considered a certified VA screening site and participated in the program. Environmental testing conditions were standardized prior to screening. Viewing distance was verified using the electronic distance measurement device Stanley TLM99s Laser Distance Measurer (Towson, MD, USA), and ambient illumination levels were measured using the digital photometric light meter Extech Lux Meter EA30 (Extech Instruments Corporation, Nashua, NH, USA) to ensure compliance with predefined testing conditions across all sites. Εxaminer-related variability was minimized through standardized equipment, automated calibration, protocol-driven testing procedures, and certification-based examiner training.

### 2.4. Data Collection

Special care was given to the consistency of the data collection. Children were examined by their teachers in the presence of a certified researcher from the program who had no direct involvement in the examination process. Since DDiVAT consists of a Smart-TV application, the vast majority of students believed that they were participating in an interactive video game. Four options were provided to the students to acknowledge that they could identify the displayed symbol on the TV screen: (a) verbal response, (b) selection of the matching symbol from a card with a set of symbols, (c) rotation of the symbol toward the correct direction, (d) pointing with his/her finger (Video S1). Upon conclusion of the measuring process, VA data automatically updated the secure cloud database, requiring no action by the teacher.

### 2.5. Assessment of Examiner-Related Variability

To evaluate potential examiner-related variability, a post hoc inter-rater reliability analysis was conducted in a randomly selected subset of 80 children. Each child underwent VA assessment using the DDiVAT system by two independent certified examiners, with the second examination performed by a different examiner under identical testing conditions. Examiners were blinded to each other’s results. The same hardware, automated calibration procedures, testing distance, optotype presentation, and standardized examination protocol were used for both assessments.

### 2.6. Statistical Analysis

Statistical analysis was performed using the Medcalc 20.1.4 statistical software package (Ostend, Belgium). The Shapiro–Wilk test was used to assess distribution. Normally distributed variables are presented as mean and 95% confidence interval, while non-normally distributed variables are presented as median and interquartile range. Comparison between variables was performed using the one-way ANOVA test when the variables were normally distributed, while the Kruskal–Wallis test was applied for variables with a non-normal distribution. For the comparison of variables evaluated at two time points during the study, the paired-sample *t*-test was used for normally distributed data, and the Wilcoxon signed-rank test for non-normally distributed data. *p*-values of less than 0.05 were regarded as statistically significant. Inter-rater reliability between examiners was assessed using the intraclass correlation coefficient (ICC) for continuous VA outcomes (logMAR). A two-way random-effects model with absolute agreement (ICC [2,1]) was applied, as both examiners were considered representative of the examiner population. ICC values were interpreted according to established thresholds, with values above 0.90 indicating excellent agreement.

## 3. Results

In the absence of internationally accepted minimal required visual acuity (mRVA) thresholds for preschool populations, age-specific cut-offs were applied in the present analysis. An mRVA of logMAR 0.3 was used for children aged ≤60 months, while an mRVA of logMAR 0.2 was applied for children older than 60 months. These thresholds were selected based on age-specific normative VA distributions reported in large population-based studies, including the Multi-Ethnic Pediatric Eye Disease Study and the Sydney Paediatric Eye Disease Study, which indicate progressive improvement in presenting visual acuity (pVA) with increasing age [10,11,12]. The selected cut-offs were intended to balance sensitivity for detecting clinically relevant visual impairment against the risk of excessive referral in a screening setting. Moreover, the better eye was defined as the eye with the better pVA, and the worse eye as the one with the worse pVA.

In the SG1 group, 84.8% of students surpassed the mRVA limit in both eyes. In addition, 94.5% surpassed mRVA only in the better eye, while 14 (1.5%) failed to reach mRVA in both eyes. Similarly, in the SG2 group, 88.3% of students surpassed the mRVA limit in both eyes, 96.4% only in the better eye, while 3.6% failed to reach mRVA in both eyes. Finally, 80.3% of SG3 students surpassed the mRVA limit in both eyes, 93.7% only in the better eye, while 1.1% failed to reach mRVA in both eyes (Table 2).

SG1 mean pVA was 0.11 logMAR in the right eye and 0.12 logMAR in the left eye. SG2 mean pVA was 0.07 logMAR in the right eye and 0.08 logMAR in the left eye, while SG3 presented pVA 0.069 logMAR in the right eye and 0.067 logMAR in the left eye, respectively. The comparison among the three groups revealed a significant improvement in pVA for older students (Table 3).

In Greece, kindergarten is a two-year program, meaning that some students were assessed over two consecutive school years. These students demonstrated significant improvement in their pVA (Table 4).

In accordance with the mRVA criteria that were set in the present study, 207 students (8.36%) were referred for further evaluation, which included the 51 who failed to complete the test. Possible reasons for suboptimal pVA and referral included: (a) refractive errors (134, 5.41%), and strabismus (20, 0.81%).

Only minor technical difficulties were encountered during the implementation of the program, attributed to local instabilities of the internet connection, which resulted in short delays during the testing. Teachers praised the user-friendliness of the application, including the automatic storing and transmission of pVA data that required no action from them, and the high familiarity of the students with the Smart-TV.

Of the 207 children referred based on the predefined mRVA criteria, 148 (71.5%) underwent comprehensive ophthalmologic examination at our department. VA impairment was confirmed in 145 of these children, yielding a positive predictive value (PPV) of 98%. Refractive errors accounted for 82.4% (122/148) of confirmed cases, while strabismus and/or amblyopia were identified in 16.2% (24/148).

Inter-rater reliability analysis demonstrated excellent agreement between examiners. In the subset of 80 children assessed independently by two different examiners, the intraclass correlation coefficient for VA measurements was ICC = 0.92, indicating minimal examiner-related variability within the DDiVAT screening framework.

## 4. Discussion

The primary objective of the present study was the implementation of a VA screening program for the preschool population in East-Northern Greece and to explore the feasibility of expanding the program on a nationwide, or even Europe-wide scale. Specifically, this study was not designed as a diagnostic accuracy study, but rather as a feasibility and implementation study of a structured screening program. Program methods relied on four fundamental principles: (a) certification of teachers as reliable data collectors, (b) consistency in the data collection process, (c) user friendliness towards the students and the teachers, and (d) automatic storage and trapping of suspicious cases. Although the present findings demonstrate that a standardized digital screening framework can be implemented across multiple kindergarten sites within a metropolitan region, broader scalability should be interpreted cautiously. The current data support feasibility at the levels of protocol deployment, user acceptance, and technical consistency; however, critical dimensions of scalability—including cost-effectiveness, infrastructure capacity, workforce requirements, and long-term sustainability—were not assessed.

The aforementioned principles were addressed as follows. All participating teachers received a 90 h, blended course on the principles of VA screening, targeted specifically at preschool-age minors. The present findings support previous evidence that school-based VA screening can be effectively delivered by non-health personnel when appropriate training and standardized tools are employed [3,13]. The high level of consistency observed across participating schools suggests that the screening outcomes were not substantially influenced by local factors, but rather reflected the uniformity of the screening protocol and the centralized digital infrastructure. This observation aligns with prior reports indicating that standardization of procedures is a critical determinant of reliability in large-scale vision screening programs [3,13]. The use of a digital, smart-TV-based screening platform may have contributed to high acceptability among both teachers and children, potentially facilitating engagement and reducing variability related to testing environment or examiner behavior. Similar benefits of digital platforms have been reported in pediatric screening contexts, where familiar interfaces and paperless workflows are associated with improved compliance and operational efficiency [14,15]. Importantly, the minimal inter-rater reliability observed in exploratory analyses supports the robustness of the screening framework and suggests that the reported outcomes are generalizable across different educational settings within the study region. Given the exploratory nature of these analyses and the absence of predefined hypotheses, school-level comparisons were not pursued further, and the Discussion focuses on overall screening performance rather than site-specific effects. Specifically, although this analysis demonstrated excellent agreement between certified examiners, it was conducted in a limited subset of participants and may not fully reflect examiner-related variability across teachers with differing levels of experience or across heterogeneous educational settings.

The age-specific mRVA thresholds applied in this study, although based on large normative datasets, remain operational choices rather than universally accepted standards. Alternative cut-offs could meaningfully influence referral rates, with more stringent thresholds likely increasing sensitivity at the expense of specificity, and more lenient thresholds reducing referrals but risking under-detection of early amblyogenic conditions. Given the preventive aim of preschool screening, the selected thresholds were intentionally conservative. Future studies incorporating outcome-based validation or sensitivity analyses across different cut-off values may help refine optimal referral criteria for this age group.

We cannot directly compare the outcomes of the present study with those of previous ones from Greece, since no locally published reports could be retrieved. However, 1.7% of our students wore spectacles, which is on the low end, compared to the international literature, which suggests variable but higher numbers that range between 1.5% (3–4 years) to 12.8% (6–7 years) [16,17]. This discrepancy may be attributed to differences in screening age ranges, referral thresholds, healthcare system organization, and access to pediatric eye care services. Additionally, cultural attitudes toward early spectacle use and the lack of a universally implemented preschool vision screening program in Greece may contribute to lower rates of early spectacle prescription. These findings underscore the importance of context-specific interpretation of screening outcomes and highlight the need for standardized national screening pathways. Average pVA ranged from logMAR 0.11 to logMAR 0.07, which was significantly better in older children, consistent with the published literature [10,18]. Regarding referral rates, it is well known that different guidelines contribute to the high variability observed in published reports [19]. Nevertheless, 8.7% of the students were referred for ophthalmological examination, which included the 2.1% that failed to cooperate.

A major limitation of the present study is the absence of systematic ophthalmological follow-up and diagnostic confirmation for referred children. Importantly, the study was not designed as a diagnostic accuracy investigation, but as a pragmatic implementation and feasibility evaluation of a public health screening and referral pathway. Consequently, statistical analyses focused on population-level distributions, group comparisons, longitudinal trends, and referral outcome confirmation rather than sensitivity and specificity estimation. Given the pragmatic screening design, incomplete outcome verification, and limited availability of individual-level covariates, multivariable modeling was not undertaken in the present analysis to avoid overinterpretation of associations. Accordingly, the primary aim was to evaluate the practicality, acceptance, and screening yield of a kindergarten-based digital VA screening framework in real-world educational settings. Future iterations of the program are planned to incorporate structured follow-up and outcome tracking, which will allow confirmation of diagnoses, estimation of false-positive and false-negative rates, and assessment of the clinical impact of the screening process. Moreover, the descriptive design and the absence of systematically collected individual-level covariates eliminate the use of multivariable analyses to explore independent predictors of reduced pVA or referral. Future iterations of the screening program will aim to incorporate additional demographic and clinical variables to enable more advanced risk modeling. Furthermore, while DDiVAT is a certified Class-1 medical device and prior studies have demonstrated its reliability and usability in screening contexts, its diagnostic accuracy parameters, such as sensitivity and specificity in preschool populations, remain to be determined [8,9]. The absence of gold-standard comparison studies limits the ability to quantify false-positive and false-negative rates in the present setting. Future research should focus on controlled validation studies comparing DDiVAT directly with standardized pediatric VA charts, enabling robust estimation of sensitivity, specificity, and predictive values in preschool vision screening. A relevant limitation of the present study is also that ophthalmologic confirmation was completed among 71.5% of referred children, introducing the possibility of verification bias. As follow-up attendance was not universal, the calculated PPV reflects diagnostic yield among children who completed the examination rather than the entire referred cohort. It is plausible that children with more evident visual difficulties or greater parental concern were more likely to attend follow-up, potentially inflating the observed PPV. Conversely, non-attendance does not necessarily imply absence of pathology, as access, socioeconomic factors, or caregiver availability may have influenced follow-up completion. Therefore, while the high PPV supports the clinical relevance of the referral criteria, it should be interpreted cautiously and not assumed to represent the absolute PPV of the screening program at the population level. Notably, despite incomplete verification, the very high confirmation rate among examined children suggests that false-positive referrals were uncommon, supporting the clinical specificity of the applied mRVA criteria.

**Figure 1:**
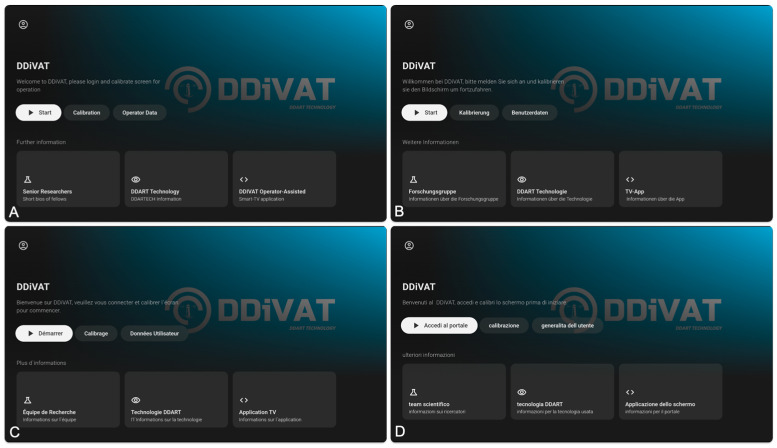
DDiVAT application: main screen for user login and calibration, shown in four different European languages (**A**) English, (**B**) German, (**C**) French, (**D**) Italian.

We performed a rudimentary Strengths Weaknesses Opportunities Threats analysis (SWOT analysis). This assessment was not intended as a formal analytical evaluation, but rather as a descriptive, hypothesis-generating overview. Given the absence of cost-effectiveness analyses, health-economic modeling, or structured resource assessment, these observations should be interpreted cautiously. From an implementation perspective, a key strength of the framework is the use of a certified Smart-TV-based application for VA screening, which enables standardized deployment across sites and multilingual adaptation. It is globally available from the Google Play Store; it senses the local settings of the TV and adapts to 15 European languages (Figure 1).

DDiVAT is supported by a cloud-based database, which has already been awarded the necessary certifications regarding the safety of its data and compliance with the General Data Protection Regulation (GDPR). Moreover, it is supported by an official multilingual, lifelong-learning training certification course offered by the Training and Lifelong Learning Center of Democritus University of Thrace, which secures the competence of teachers as reliable data collectors in VA screening. On the other hand, the weaknesses are few and limited to the fact that DDiVAT is validated as a distance VA test and cannot simulate other comprehensive school-based examinations for a complete ophthalmological screening. The opportunities for a Europe-wide preschool program are significant. There is an unmet need for a pan-European registry of VA for the preschool population, since it is estimated that in the following years, the prevalence of refractive errors and other ophthalmological faults will escalate [20]. On the other hand, among the primary threats is the fact that there is no consensus among European Union member countries on the methods of measuring pVA in preschool children and on the mRVA according to the students’ age.

## 5. Conclusions

In conclusion, we report on the methods and on the outcomes of the first official preschool VA screening program in Greece. Mean presented VA scores and referral rates are consistent with published reports from other Western countries.

Overall, while the operational feasibility, user acceptance, and screening yield observed in this study indicate that the DDiVAT framework can be integrated into kindergarten-based screening workflows, these findings should be interpreted cautiously. The absence of systematic follow-up, cost-effectiveness analyses, and health-economic evaluation underscores the need for independent, evidence-based studies before broader implementation can be considered. Nevertheless, the present findings suggest that the DDiVAT screening framework may represent a feasible digital approach for preschool VA screening in real-world educational settings. Its potential role in larger-scale screening initiatives remains conditional upon future studies establishing standardized referral criteria, incorporating economic evaluation, and assessing longer-term clinical outcomes.

## Figures and Tables

**Table 1:** Study participants.

Parameters	Values
Students (N)	2476
Boys/Girls (N)	1256/1220
Age (months) (Mean ± SD), [Range]	62.98 ± 7.54 [36–84]
SG1 (N)	962
SG2 (N)	1194
SG3 (N)	269
Fail to cooperate (N)	51

N: number of participants, SD: Standard Deviation, SG1: Study Group 1, SG2: Study Group 2, SG3: Study Group 3.

**Table 2:** Presenting visual acuity.

	SG1 (36–60 Months)	SG2 (61–72 Months)	SG3 (≥73 Months)
Students with pVA equal or better mRVA in both eyes	816 (84.82%)	1054 (88.27%)	216 (80.30%)
Students with pVA equal or better mRVA in better eye	909 (94.49%)	1151 (96.40%)	252 (93.68%)
Students with pVA worse than mRVA in both eyes	14 (1.46%)	43 (3.60%)	3 (1.12%)
Students with logMAR pVA >0.20 to ≤0.30	N/A	20 (1.68%)	1 (0.37%)
Students with logMAR pVA >0.30 to ≤0.40	4 (0.42%)	4 (0.34%)	1 (0.37%)
Students with logMAR pVA >0.40 to ≤0.50	1 (0.10%)	0 (0.00%)	0 (0.00%)
Students with logMAR pVA >0.50	2 (0.21%)	2 (0.17%)	1 (0.37%)
Total	962	1194	269

**Table 3:** Comparison of presenting visual acuity.

Group	N	VA OD [95%CI]	VA OS [95%CI]	*p* Value OD		*p* Value OS	
SG1	962	0.11 [0.10–0.12]	0.12 [0.11–0.12]	*p* < 0.001 * (Group 2)	*p* < 0.001 * (Group 3)	*p* < 0.001 * (Group 2)	*p* < 0.001 * (Group 3)
SG2	1194	0.07 [0.07–0.08]	0.08 [0.07–0.09]	*p* < 0.001 * (Group 1)	*p* = 0.67 (Group 3)	*p* < 0.001 * (Group 1)	*p* = 0.20 (Group 3)
SG3	269	0.07 [0.06–0.08]	0.07 [0.05–0.08]	*p* < 0.001 * (Group 1)	*p* = 0.67 (Group 2)	*p* < 0.001 * (Group 1)	*p* = 0.20 (Group 2)

* *p* < 0.001, CI: confidence interval, N: number of participants, OD: right eye, OS: left eye, VA: visual acuity.

**Table 4:** Participants examined over two consecutive school years.

School Year	N	VA OD 2022–2023 [IQR] (logMAR)	VA OS 2022–2023 [IQR] (logMAR)	VA OD 2023–2024 [IQR] (logMAR)	VA OS 2023–2024 [IQR] (logMAR)	VA OD 2024–2025 [IQR] (logMAR)	VA OS 2024–2025 [IQR] (logMAR)	*p* Value	
2022–2023 vs. 2023–2024	243	0.10 [0.02, 0.14]	0.10 [0, 0.18]	0.04 [0.02, 0.06]	0.04 [0, 0.10]	NA	NA	<0.001 * (OD)	0.001 * (OS)
2023–2024 vs. 2024–2025	148	NA	NA	0.10 [0.04, 0.18]	0.10 [0, 0.16]	0.02 [0, 0.10]	0.06 [0, 0.10]	<0.001 * (OD)	0.009 * (OS)

* *p* < 0.05, IQR: interquartile range, NA: not applicable, OD: right eye, OS: left eye, VA: visual acuity.

## Data Availability

The data supporting the findings of this study are available from the corresponding author upon reasonable request. Due to ethical and privacy considerations, certain restrictions may apply to the availability of the data.

## Supplementary Materials

The following supporting information can be downloaded at: https://www.mdpi.com/article/10.3390/jcm15051907/s1, Video S1: Identification methods of TV-displayed symbols.

## Abbreviations

The following abbreviations are used in this manuscript:

DDiVAT	Democritus Digital Visual Acuity Test
VA	Visual Acuity
SG1	Study Group 1
SG2	Study Group 2
SG3	Study Group3
mRVA	Minimal Required Visual Acuity
pVA	Presenting Visual Acuity

## References

[1] UNESCO, UNICEF, World Food Programme (2023). Ready to Learn and Thrive: School Health and Nutrition Around the World.

[2] Congdon N., Chan V.F. (2023). Schools, children and myopia. Am. J. Ophthalmol..

[3] Liu S.M., Chang F.C., Chen C.Y., Shih S.-F., Meng B., Ng E., Hsu C.-H., Chiang Y.-T., Mao X.-J., Yi M.-Y. (2021). Effects of parental involvement in a preschool-based eye health intervention regarding children’s screen use in China. Int. J. Environ. Res. Public Health.

[4] Sharma A., Li L., Song Y., Choi K., Lam D.S.C., Zhang M., Zheng M., Zhou Z., Liu X., Wu B. (2008). Strategies to improve the accuracy of vision measurement by teachers in rural Chinese secondary schoolchildren: Xichang Pediatric Refractive Error Study (X-PRES) report no. 6. Arch. Ophthalmol..

[5] Alrasheed S. (2023). Systematic review and meta-analysis of childhood visual impairment in the Eastern Mediterranean Region. East. Mediterr. Health J..

[6] Toledo C.C., Paiva A.P., Camilo G.B., Maior M.R., Leite I.C., Guerra M.R. (2010). Early detection of visual impairment and its relation to academic performance. Rev. Assoc. Med. Bras..

[7] Jonas D.E., Amick H.R., Wallace I.F., Feltner C., Schaaf E.B.V., Brown C.L., Baker C. (2017). Vision screening in children aged 6 months to 5 years: Evidence report and systematic review for the US Preventive Services Task Force. JAMA.

[8] Labiris G., Delibasis K., Panagiotopoulou E.K., Pigadas V., Bakirtzis M., Panagis C., Dardabounis D., Ntonti P. (2022). Development and Validation of the First Smart TV-Based Visual Acuity Test: A Prospective Study. Healthcare.

[9] Bakirtzis M., Michaleakou E., Martidou M.E., Lahana E., Kostagiolas P., Niakas D., Labiris G. (2024). Visual acuity screening of refugees and immigrants with a web-based digital test: A pilot study. Acta Med..

[10] Pai A.S., Wang J.J., Samarawickrama C., Burlutsky G., Rose K.A., Varma R., Wong T.Y., Mitchell P. (2011). Prevalence and risk factors for visual impairment in preschool children: The Sydney Paediatric Eye Disease Study. Ophthalmology.

[11] Guo X., Fu M., Lü J., Chen Q., Zeng Y., Ding X., Morgan I.G., He M. (2015). Normative distribution of visual acuity in 3- to 6-year-old Chinese preschoolers: The Shenzhen Kindergarten Eye Study. Investig. Ophthalmol. Vis. Sci..

[12] Pan Y., Tarczy-Hornoch K., Cotter S.A., Wen G., Borchert M.S., Azen S.P., Varma R. (2009). Visual acuity norms in preschool children: The Multi-Ethnic Pediatric Eye Disease Study. Optom. Vis. Sci..

[13] Little J.A., Chan V.F., Saw S.M., Tham Y.C., Chew L., Foo L.L., Collins M., Ebri A.E., Han X., Schultz L. (2025). Current status of school vision screening—Rationale, models, impact, and challenges: A review. Br. J. Ophthalmol..

[14] Carson V., Tremblay M.S., Spence J.C., Timmons B.W., Janssen I. (2013). The Canadian sedentary behaviour guidelines for the early years (zero to four years of age) and screen time among children from Kingston, Ontario. Paediatr. Child Health.

[15] Carson V., Langlois K., Colley R. (2020). Associations between parent and child sedentary behaviour and physical activity in early childhood. Health Rep..

[16] Iyer V., Enthoven C.A., van Dommelen P., van Samkar A., Groenewoud J.H., Jaddoe V.V.W., Reijneveld S.A., Klaver C.C.W. (2022). Rates of spectacle wear in early childhood in the Netherlands. BMC Pediatr..

[17] O’Donoghue L., McClelland J.F., Logan N.S., Rudnicka A.R., Owen C.G., Saunders K.J. (2010). Refractive error and visual impairment in school children in Northern Ireland. Br. J. Ophthalmol..

[18] Sanker N., Dhirani S., Bhakat P. (2013). Comparison of visual acuity results in preschool children with Lea symbols and Bailey-Lovie E chart. Middle East Afr. J. Ophthalmol..

[19] Sechrist S.J., de Alba Campomanes A.G. (2024). The effect of inconsistent guidelines on variability in pediatric vision screening referral outcomes. J. Am. Assoc. Pediatr. Ophthalmol. Strabismus.

[20] Liang J., Pu Y., Chen J., Liu M., Ouyang B., Jin Z., Ge W., Wu Z., Yang X., Qin C. (2025). Global prevalence, trend, and projection of myopia in children and adolescents from 1990 to 2050: A comprehensive systematic review and meta-analysis. Br. J. Ophthalmol..

